# Comprehensive Characterization of Fruit Volatiles and Nutritional Quality of Three Cucumber (*Cucumis sativus* L.) Genotypes from Different Geographic Groups after Bagging Treatment

**DOI:** 10.3390/foods9030294

**Published:** 2020-03-05

**Authors:** Nan Shan, Zengyu Gan, Jing Nie, Huan Liu, Zhenyu Wang, Xiaolei Sui

**Affiliations:** 1 Beijing Key Laboratory of Growth and Developmental Regulation for Protected Vegetable Crops, College of Horticulture, China Agricultural University, Beijing 100193, China; shannan@jxau.edu.cn (N.S.); ganzy@jxau.edu.cn (Z.G.); niejing0606@163.com (J.N.); dempseyliuhuan@cau.edu.cn (H.L.); 2 College of Agronomy, Jiangxi Agricultural University, Nanchang 330045, China; 3 Beijing High-Quality Agro-Products Service Station, Beijing 100101, China; wangzhenyu1987565@163.com

**Keywords:** cucumber, bagging, hydroperoxide lyase, lipoxygenase, flavor quality, volatile compounds

## Abstract

Bagging is widely practiced to produce high quality and unblemished fruit; however, little is currently known about the effect of bagging on flavor and nutritional quality of cucumber fruits. Here we determined the influence of bagging on fruit quality of cucumber (*Cucumis sativus* L.) using three genotypes from different geographic groups. Exocarp chlorophyll and carotenoid levels were significantly decreased by bagging, accompanied by color change. Ascorbate content in bagged fruits decreased to some extent, while contents of soluble sugars, starch, and cellulose were comparable with those of control fruits. Compositions related to fruit flavor quality could be enhanced largely through bagging treatment, with elevation of the relative proportion of C_6_ aldehyde, as well as (*E,Z*)-2,6-nonadienal/(*E*)-2-nonenal ratio, and linoleic/α-linolenic acid ratio. Lipoxygenase and hydroperoxide lyase, two key enzymes in the production of volatiles, displayed distinctive transcript expression patterns and trends in changes of enzymatic activity in the bagged fruits of different genotypes. Overall, this study assesses the information on changing characteristics of fruit volatile composition and nutritional quality among different cucumber genotypes after bagging treatment. Results of this study would contribute to providing reference for mechanism study and cultivation conditions to improve cucumber fruit flavor to a considerable degree.

## 1. Introduction

Cucumber (*Cucumis sativus* L.) is a widely grown vegetable with a fresh and distinct flavor. Researchers have long worked to improve the commercial qualities of cucumbers, which—like other fruit and vegetable agricultural products—are evaluated externally based on color, texture, size, shape, and visual defects [1,2]. Fruit color is determined by the interaction of pigment molecules, such as chlorophyll (Chl) and carotenoids [3]. Chlorophyll, a tetrapyrrole compound present in higher plants and all other photosynthetic organisms, contributes to the green color. Carotenoids are ubiquitous pigments that confer yellow, orange-red, or red colors to higher plants, fungi, and algae. The biosynthesis of both chlorophyll and carotenoids is tightly regulated by light [4]. The chlorophyll b transiently increased in the shade treatments at 5 days, with the extension of shading time, and the relative chlorophyll a decreased significantly [5]. Bagging significantly reduced chlorophyll and carotenoid content in fruit flesh of kiwi (*Actinidia eriantha*) [6]. 

Fruit nutritional and flavor characteristics are important internal components of quality. Soluble sugars, such as glucose and fructose, as well as insoluble sugars, including cellulose and starch, contribute to both nutritional composition and texture of fruits. Flavor is defined as the combination of taste and odor. Almost 78 volatile compounds related to flavor have been identified from cucumber fruits, including aldehydes, alcohols, esters, alkanes, furfurans, and others, with aldehydes and alcohols accounting for a large proportion of these [7].

**Figure 1:**
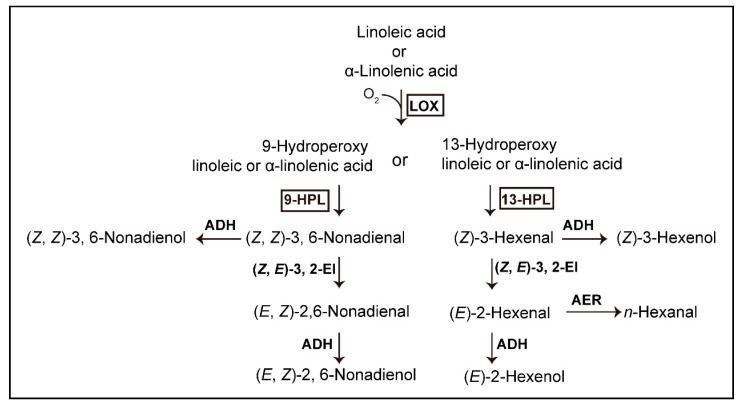
Biosynthesis of selected flavor compounds derived from fatty acids (modified from Schwab et al., (2008) [11]). ADH, alcohol dehydrogenase; AER, alkenal oxidoreductase; HPL, hydroperoxide lyase; LOX, lipoxygenase; (Z, E)-3, 2-EI, (Z, E)-3, 2-enal isomerase.

Volatile C_6_ and C_9_ aldehydes tend to be important contributors to flavor in fruits and vegetables [8,9,10]. (*E,Z*)-2,6-nonadienal, which has a cucumber-like flavor, and (*E*)-2-hexenal, which has an apple-like flavor, are the main volatile compounds in cucumber fruits [7]. C_6_ and C_9_ compounds are produced through the lipoxygenase (LOX) and hydroperoxide lyase (HPL) pathways [7,10]. LOX and HPL are key enzymes in the oxylipin pathway encoded by multiple gene families [11]. LOX catalyzes the stereospecific oxidation of polyunsaturated fatty acids such as linoleic acid and α-linolenic acid. Plant LOXs are classified with respect to their positional specificity of fatty acid oxygenation, which can occur either at C_9_ (9-LOX) or at C_13_ (13-LOX) of the hydrocarbon backbone in the case of a C_18_ fatty acid [12]. The *LOX* gene family in cucumber has 23 members. Generally, they can be divided into two types, i.e., type-1 and type-2, which are involved in the biosynthesis of C_9_ aldehydes and C_6_ aldehydes, respectively [13,14]. Several type-1 and type-2 *LOX* genes have also been found to be expressed primarily in fruits [15]. The hydroperoxides produced by 13- or 9-LOX are subsequently cleaved by 13-HPL into C_6_ aldehydes or by 9-HPL into C_9_ aldehydes, respectively (Figure 1). Three HPL enzymes in cucumber are divided into three types according to their substrate specificities: 13-HPL (encoded by *CsHPL1*) that specifically catalyzes 13-hydroperoxide into C_6_ volatiles, 9-HPL (*CsHPL3*) that catalyzes 9-hydroperoxides to form C_9_ aldehydes, and 9/13-HPL (*CsHPL2*) that can use both 9- and 13-hydroperoxides as substrates to produce C_6_ and C_9_ aldehydes [16].

Fruit nutritional and flavor qualities are also influenced by other intrinsic (such as cultivar and stage of maturity) and imposed (for example, environment, cultural techniques, and storage) factors [8,17]. Light, temperature, oxygen, and ethylene all influence volatile metabolism [17]. Fruit bagging is applied to improve fruit quality by altering its exposure to light and is a useful method for studying fruit quality during production [8,17,18]. In this study, we used black paper bags to shade the fruit of different cucumber genotypes on the day of anthesis. The effects of bagging on apparent color, nutritional quality, and volatiles variation of fruits were assessed. The present study will provide reference for mechanism study and cultivation conditions such as bagging that probably improve cucumber fruit flavor to a considerable degree.

## 2. Materials and Methods 

### 2.1. Plant Materials, Growth Conditions, and Treatments

Three cucumber genotypes “Zhongnong No.16” (“ZN16”, from the Institute of Vegetables and Flowers, Chinese Academy of Agricultural Sciences, belongs to East Asian group), “Baiyesan” (“BYS”, belongs to East Asian group), and “Deltastar” (“DS”, from Rijk Zwaan, belongs to Eurasian group) were grown in a growth chamber with a 10 h photoperiod and a temperature cycle of 25/18 °C (day/night). The photon flux density was 28 kilolux. Seedlings were transferred to a solar greenhouse and arranged in a completely randomized design with three replicates. All three genotypes belong to the slicing type for fresh eating. Bagging treatment was conducted according to a previous study [19]. On the day of anthesis, half of the ovaries were bagged with a double-layer black kraft paper bag (0.0804 mm thick, density 0.0588 kg·m^−2^) to exclude light, with non-bagged fruits as a control. Both bagged and non-bagged fruits were harvested at 9 d after anthesis/bagging (DAA/DAB).

### 2.2. Determination of Photosynthetic Pigments, Ascorbate, Sugar, Cellulose, and Starch

Chlorophyll (Chl) and carotenoid contents were measured according to standard methods [19]. Briefly, Chl and carotenoid were extracted using 80% (*v/v*) acetone, and the spectrophotometric absorbance of supernatant at 645 nm, 663 nm and 652 nm were used to calculate Chl and carotenoid content. 

Ascorbate content was estimated as described previously [20]. Briefly, cucumber fruits were homogenized with 5% (*w/v*) trichloroacetic acid solution. After centrifugation at 2300× *g* for 10 min, supernatants were mixed with ethyl alcohol (EtOH), followed by 0.4% (*v/v*) H_3_PO_4_-EtOH, 0.5% (*w/v*) bathophenanthroline (BP)-EtOH, and finally 0.03% (*w/v*) FeCl_3_-EtOH. The solution was kept at 30 °C for 90 min for a Fe^2+^-BP complex to develop. The absorbance of the colored solution was read at 534 nm with a spectrophotometer. 

Sugars, cellulose, and starch were extracted as described previously [21]. Soluble sugars, including fructose, glucose, and sucrose were extracted using 80% (*v/v*) ethanol. High-performance liquid chromatography (Agilent 2100 system, Agilent Technologies, Palo Alto, CA, USA) was used to measure and analyze sugar content. Residues were treated with 30% (*v/v*) perchloric acid for starch extraction. For measurement of cellulose, residues were further treated three times with 1 mol L^−1^ NaOH, hot water, and acetone, successively, for 15 min each, followed by 60% (*v/v*) sulfuric acid for 16 h at 4 °C three times. The contents of starch and cellulose in the supernatant were determined using the anthrone-sulfuric acid colorimetric method at 620 nm with a spectrophotometer [22].

### 2.3. Analysis of Volatile Compounds

Volatile measurement was conducted by solid-phase microextraction (SPME) combined with gas chromatography (GC)-mass spectrometry (MS) according to previous methods [7,23,24,25] with modifications. Fresh cucumber fruit was ground to power and transferred to a 15 mL vial, followed by addition of 1.5 mL saturated sodium chloride solution to disrupt the activity of endogenous enzymes and increase the solution’s ionic strength. The volatile compound octanal was added as an internal standard [7]. The vial was then tightly sealed with screw cap containing PTFE-coated silicon septa (Gerstel), then vortexed for 30 s. Each sample was extracted at 35 °C for 30 min.

A 100 μm fused silica fiber coated with PDMS (Sigma-Aldrich) was used to collect a wide range of volatile compounds from the headspace [26,27]. After being preheated at 35 °C for 5 min, the fiber was inserted into the vial and exposed to the headspace at 35 °C for 30 min. Volatile compounds were measured using a Shimadzu GCMS-QP2010 Plus mass spectrometer (Shimadzu Corporation, Kyoto, Japan) on a DB-5MS capillary column (30 m × 0.25 mm × 0.25 μm). GC conditions: helium as carrier gas and flow rate of 1 mL min^−1^. The injector temperature was set at 250 °C. To facilitate separation and elution of the injected headspace volatile compounds, the initial oven temperature was 60 °C for 2 min, which increased to 220 °C at a speed of 8 °C min^-1^ and was held for 20 min. MS conditions: ion source temperature of 200 °C, electron ionization with an electron energy of 70 eV, a scanning range of 50 to 400 *m/z*. 

The peaks of aromatic components were identified and purified by automated mass spectral deconvolution and identification system combined with retention index (RI), then further processed by Compound Composer software to eliminate background peaks. Volatile compounds were further identified by matching and reverse matching with the NIST mass spectral library (National Institute of Standards and Technology, USA) of no less than 90%, comparing spectra and retention times with commercially available standards, and comparison of experimental retention index with RI according to literature [7,23,25,28]. The peak area of each compound was normalized to the peak area of octanal prior to further data processing. Odor threshold, which is the lowest concentration that can be perceived by human olfaction, was identified based on previous study [7] and presented in Table 1.

### 2.4. RNA Isolation, Reverse-Transcription Quantitative PCR Analysis, and Measurement of LOX and HPL Activities

Total RNA was isolated from cucumber fruits using a Quick RNA isolation kit (Huayueyang, China) according to the manufacturer’s instructions. After removal of potential genomic DNA using RNase-free DNase I, purified RNAs were reverse transcribed into cDNA using a PrimeScript First Strand cDNA Synthesis kit (TaKaRa, Japan). Primers specific for cucumber *HPL* and *LOX* genes were synthesized in a previous study [13,15]. PCR products were amplified using an Applied BioSystems 7500 Real Time PCR System (Applied Biosystems, Foster City, CA, USA) with a SYBR^®^ Premix Ex Taq kit (TaKaRa, Japan). Three biological replicates with three technical replicates were performed for each reverse-transcription/PCR procedure. For relative quantification, the cucumber *α-TUBULIN* gene was the internal control [29]. Relative expression levels were calculated according to the 2^–ΔΔC^_T_ method [30]. Enzymatic activities of LOX and HPL were measured as described previously [7,31]. Total soluble protein content was determined according to the method of Sui et al. [19].

### 2.5. Measurement of Fatty Acids

Fatty acids were measured using accelerated solvent extraction (ASE) with GC-MS with modifications [32]. Cucumber fruit (~20 g) was vacuum freeze-dried at −55 °C, then crushed into powder in a mortar. Dry power was extracted in petroleum ether with a Dionex^™^ ASE^™^ 300 ASE system (USA) at 125 °C and 10.34 MPa for 25 min. The solvent was removed by a rotary evaporator to obtain the lipid. After dissolving the lipid in isooctane, a solution containing KOH (2 mol L^−1^) dissolved in methanol was added, and the sealed tube was oscillated for 30 s to produce fatty acid methyl esters. Then, anhydrous sodium sulfate was added and inverted to remove excess water. The supernatant was collected and filtered with a 0.22 μm filter membrane for GC-MS analysis. Fatty acids were measured by GC-MS system (GCMS-QP2010, Shimadzu Corporation, Japan) on a Supelco SP 2560 column (100 m × 0.25 mm × 0.20 μm). GC conditions were set as follows: inlet temperature of 250 °C, carrier gas flow rate of 1.14 mL min^−1^, total flow rate of 15.5 mL min^−1^, split ratio 10:1. The sample volume was 1 μL. To ensure good separation of fatty acid methyl esters, the initial temperature was 50 °C for 2 min, which increased to 210 °C at 4 °C min^−1^, and then to 240 °C at 1 °C min^−1^. MS conditions were set as follows: ion source temperature of 200 °C, interface temperature of 250 °C, and detector voltage of 0.1 kV. Reference standards of methyl linoleate and methyl linolenate were purchased from Sigma-Aldrich (St. Louis, MO, USA), and isooctane was used to prepare standard solutions with different concentrations. The standard curves of methyl linoleate and methyl linolenate were drawn with different concentration as the abscissa and the corresponding peak area as the ordinate. The contents of linoleic acid and α-linolenic acid in cucumber fruits were calculated based on the standard curve accordingly.

### 2.6. Statistical Analysis

All data were subjected to ANOVA assessed by Tukey’s test using SPSS statistical software version 20.0 (IBM, Armonk, NY, USA). Data are presented as mean ± SD. Heat map generation and hierarchical cluster analysis were carried out using R [33].

## 3. Results

### 3.1. Appearance and Nutritional Quality of Bagged Cucumber Fruits

**Figure 2:**
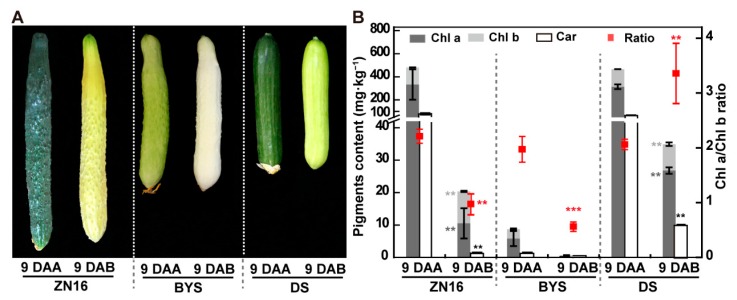
Changes in appearance quality of darkening cucumber fruits. (**A**) The color change in fruit exocarp of different cucumber genotypes at 9 DAA/9 DAB. Bar = 1 cm. (**B**) Pigments (chlorophyll and carotenoid) content based on per unit fresh weight in fruit exocarp of different cucumber fruits at 9 DAA/9 DAB. The red points in (**B**) represent the Chl a/Chl b ratio indicated by the Y-axis on the right. Error bars represent the standard deviation, *n* = 3. Significant differences were assessed by Tukey’s test (** *P* < 0.01, *** *P* < 0.001). Car, carotenoid; Chl, chlorophyll; DAA, days after anthesis; DAB, days after bagging.

We used three cucumber genotypes, “ZN16”, “BYS”, and “DS”, with distinct colors to study the effect of light environment on the appearance (color) and nutrition of fruits (Figure 2A). After bagging for 9 d (marketable maturity), the chlorophyll (Chl) and carotenoid contents in the exocarp of all cucumber fruit decreased significantly compared with those of control fruit (Figure 2B), accompanied by color changes (Figure 2). The exocarp of dark green “ZN16” and green “DS” both turned yellowish green, meanwhile, “BYS” turned from whitish green to completely white (Figure 2A). In particular, when compared with control fruits, the exocarp parenchyma cells of darkened “ZN16” and “BYS” fruit showed a decreased Chl a/b ratio due to the relatively lower Chl a and higher Chl b content of fruits under the darkened environment (Figure 2B). This indicated that bagging blocks illumination of the cucumber fruit surface, thereby inducing color changes (Figure 2).

**Figure 3:**
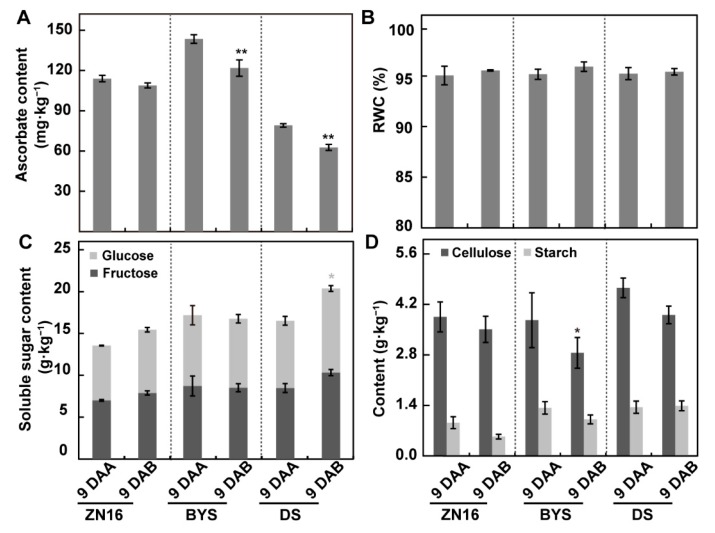
Changes in fruit nutritional quality after darkening treatment. Ascorbate content (**A**), relative water content (RWC) (**B**), soluble sugars (fructose and glucose) content (**C**), and starch and cellulose content (**D**) of fruits at 9 DAA/9 DAB. Bars represent means ± standard deviations (*n* = 3); significant differences were assessed by Tukey’s test (** *P* < 0.01, * *P <* 0.05). The light grey asterisk in (**C**) indicates a significant difference in glucose content.

We measured ascorbate, soluble sugar (fructose and glucose), starch, and cellulose contents to evaluate the nutritional value and flavor of cucumber fruits (Figure 3). Ascorbate (Figure 3A) and cellulose (Figure 3D) contents were somewhat affected by the dark growing environment, showing a little decrease in specific genotypes, whereas relative water content (Figure 3B), soluble sugar (Figure 3C) and starch content (Figure 3D) remained almost unchanged.

### 3.2. Analysis of Cucumber Fruit Volatiles and Their Variation

**Figure 4:**
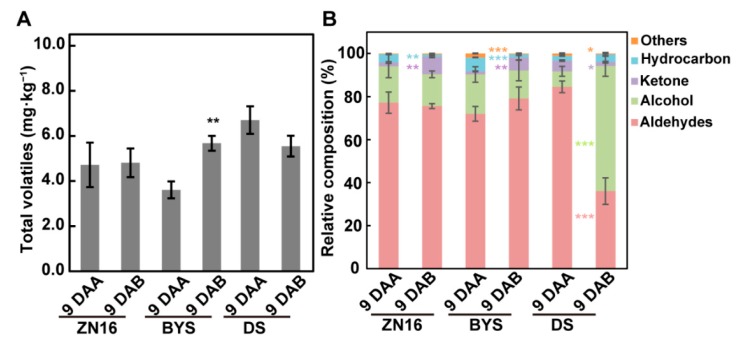
Total volatile analysis in cucumber fruits after darkening treatment. The data set for volatile analysis is given in Supplementary Table S1. Total volatile content (**A**) and composition (**B**) in cucumber fruits after bagging treatment were analyzed by ANOVA followed by Tukey’s test (* *P* < 0.05, ** *P* < 0.01, *** *P* < 0.001), and bars represent means ± standard deviations (*n* = 3). Values are means of three independent biological replicates.

The dark environment changed the volatile content and relative composition (Figure 4, Supplementary Table S1) of cucumber fruits. Among the volatiles, the aldehydes constituted the main class (from 74% to 82%), followed by alcohols (7.2% to 18.4%), ketones (1.5% to 5.2%), hydrocarbons (2.3% to 6.5%), and other compounds (0.2% to 1.8%) (Figure 4B). This is similar to previous studies that identified aldehydes, as well as alcohols, as the main compounds in cucumber fruits [7,15]. In this study, the total volatiles content of “BYS” bagged fruits was significantly increased compared with those of control fruits (Figure 4A), accompanied with an increased percentage of aldehydes and a decreased percentage of alcohols (Figure 4B). Conversely, a sharp decline in the proportion of aldehydes and a dramatic increase in the percentage of alcohols occurred in “DS” bagged fruits (Figure 4B). These results suggested that the two main types of volatiles, aldehydes and alcohols, responded differently to the dark environment in fruit of the three genotypes, especially in “DS” fruits.

**Figure 5:**
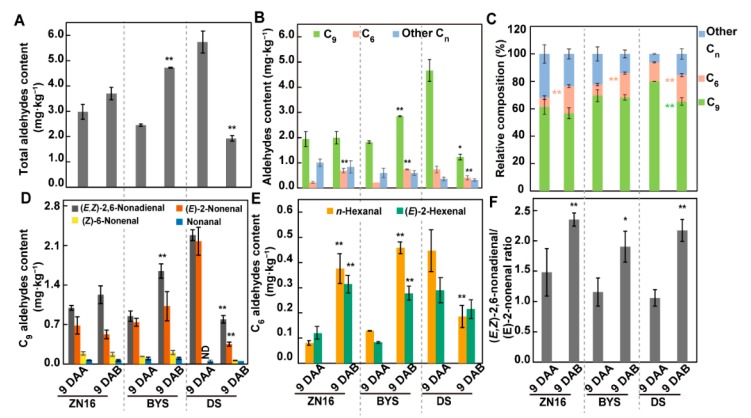
Contents and types of aldehyde compounds in cucumber fruits. (**A**) Total content of aldehydes in cucumber fruits on a fresh weight basis. Aldehydes are divided into C_6_, C_9_, and other carbon number (C_n_) aldehydes according to carbon number (**B**, **C**, Supplementary Table S1). C_9_ aldehydes comprise (*E,Z*)-2,6-nonadienal, (*E*)-2-nonenal, (*Z*)-6-nonenal, and nonenal (**D**, Supplementary Table S1), and C_6_ aldehydes consist of *n*-hexanal and (*E*)-2-hexenal (**E**, Supplementary Table S1). (**F**) Ratio of (*E,Z*)-2,6-nonadienal to (*E*)-2-nonenal content. Bars represent means ± standard deviations (*n* = 3); data were analyzed by one-way ANOVA followed by Tukey’s test (** *P* < 0.01, * *P* < 0.05). Values are means of three independent biological replicates. Pink and light green asterisks in (**C**) represent a significant difference in C_6_ and C_9_ aldehydes, respectively.

We further examined the two main compounds, aldehydes and alcohols, in cucumber fruits. “BYS” showed a significant increase in total aldehyde content in bagged fruits compared with control fruits (Figure 5A); while there is relatively little effect of bagging on the total aldehyde content of other two genotypes, “ZN16” and “DS”. Both “ZN16” and “BYS” showed an increasing trend in C_9_ aldehydes (Figure 5B) such as (*E,Z*)-2,6-nonadienal (Figure 5D) and C_6_ aldehydes (Figure 5B) such as *n*-hexanal and (*E*)-2-hexenal (Figure 5E). By contrast, total aldehydes (Figure 5A) as well as C_9_ and C_6_ aldehyde contents (Figure 5B) displayed significantly reduced levels in bagged fruits of “DS” compared with controls, mostly due to the obvious decrease of the C_9_ aldehydes (*E,Z*)-2,6-nonadienal and (*E*)-2-nonenal (Figure 5D), and the C_6_ aldehyde *n*-hexanal (Figure 5E).

Moreover, compared with control fruits, the relative proportion of C_6_ aldehydes increased significantly in all three genotypes (Figure 5C). Both (*E,Z*)-2,6-nonadienal and (*E*)-2-nonenal have been identified as aroma impact compounds in cucumber fruits [10]. The higher the (*E,Z*)-2,6-nonadienal/(*E*)-2-nonenal ratio, the stronger the fresh cucumber-like flavors [7]. This ratio was similar among “ZN16”, “BYS”, and “DS” fruits at 9 DAA, however, it increased in all genotypes after bagging, indicating probably stronger cucumber-like flavor (Figure 5F).

**Figure 6:**
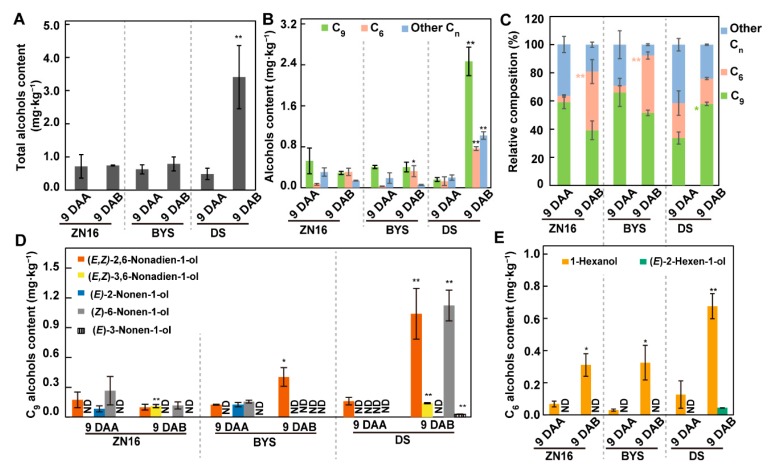
Content and type of alcohols in cucumber fruits. (**A**) Total content of alcohols in cucumber fruits. Alcohols are divided into C_6_, C_9_, and other carbon number (C_n_) alcohols according to carbon number (**B, C,** Supplementary Table S1). C_9_ alcohols comprise (*E,Z*)-2,6-nonadien-1-ol, (*E,Z*)-3,6-nonadien-1-ol, (*E*)-2-nonen-1-ol, (*Z*)-6-nonen-1-ol, and (*E*)-3-nonen-1-ol (**D**, Supplementary Table S1), and C_6_ alcohols consist of 1-hexanol and (*E*)-2-hexen-1-ol (**E**, Supplementary Table S1). Bars represent means ± standard deviations (*n* = 3); data were analyzed by one-way ANOVA followed by Tukey’s test (** *P* < 0.01, * *P* < 0.05). Values are means of three independent biological replicates. Pink and light green asterisks in (**C**) represent significant differences in C_6_ and C_9_ alcohols, respectively.

Bagging had different effects on alcohols in fruits of different genotypes (Figure 6). The levels of total alcohols, C_6_ and C_9_ alcohols, and relative percentage of C_9_ alcohols were significantly higher in bagged “DS” fruits compared with control fruits (Figure 6A–C), as were the contents of the C_9_ alcohols (*E,Z*)-2,6-nonadien-1-ol, and (*Z*)-6-nonen-1-ol (Figure 6D), as well as the C_6_ alcohol 1-hexanol (Figure 6E). Conversely, darkening treatment had little impact on total alcohols (Figure 6A) or C_9_ alcohols (Figure 6B and 6D) in “ZN16” or “BYS” 9 DAB fruits, but was accompanied by a greater relative percentage of C_6_ alcohols (Figure 6C), especially a dramatic increase in 1-hexanol content compared with controls (Figure 6E).

In general, the effects of volatile compounds on cucumber flavor are dependent on the odor threshold content, which is the lowest concentration that can be perceived by the human sense of smell, this gives rise to the aroma value, which is the ratio between volatile content and threshold content [7]. Volatile compounds with aroma values >1 have a great effect on cucumber flavor, and the higher the aroma value, the greater the influence on flavor. We further analyzed ten aroma impact compounds. Most showed increased aroma values in “ZN16” and “BYS” bagged fruits compared with control fruits, but decreased aroma values in “DS” bagged fruits (Table 1). Based on aroma value, we observed (*E,Z*)-2,6-nonadienal to be the most important odor compound, followed by (*E*)-2-nonenal (Table 1), suggesting these two compounds most likely contribute significantly to the flavor quality of cucumber fruits.

### 3.3. Gene Expression, Enzymatic Activities, and Substrate Analysis of the LOX and HPL Pathways

**Figure 7:**
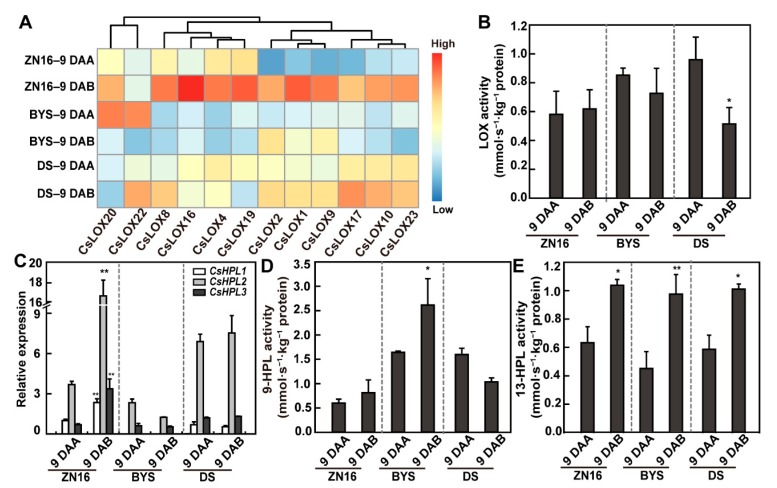
Gene expression and enzyme activities of LOX and HPL in cucumber fruits. (**A** and **C**) The expression of *CsLOX* (**A**) and *CsHPL* genes (**C**). The hierarchical cluster dendrogram and heatmap in (**A**) were generated using log10 values of gene expression levels, and color scale for fold-change values was shown. (**B, D, E**) Enzyme activities of LOX (**B**) and HPL (**D** and **E**). Enzymatic activities in (**B**), (**D**), (**E**) were calculated per kilogram protein. Bars represent means ± standard deviations (*n* = 3); data were analyzed by one-way ANOVA followed by Tukey’s test (** *P* < 0.01, * *P* < 0.05). Accession numbers of the gene sequences used for expression analysis are listed in Supplementary Table S2.

After darkening treatment, expression of most of the *CsLOX* genes was up-regulated in 9 DAB fruits of “ZN16”, “BYS” and “DS” (Figure 7A). Nevertheless, LOX enzyme activity level had no significant change in “ZN16” and “BYS” bagged fruits compared with control fruits (Figure 7B), but it was reduced to a certain degree in “DS” bagged fruits (Figure 7B), possibly resulting in the lower content of aldehydes relative to “DS” control fruit (Figure 5A–B, Figure 5D–E).

During bagging treatment, *CsHPL* genes were all up-regulated in “ZN16” 9 DAB fruits but remained unchanged in both “DS” and “BYS” bagged fruits (Figure 7C). On the one hand, among the three genotypes, only “BYS” bagged fruits showed higher 9-HPL enzyme activity (Figure 7D), corresponding to higher levels of C_9_ aldehydes in “BYS” 9 DAB fruits (Figure 5B,D); on the other hand, 13-HPL enzyme activity was increased in all genotypes by darkening treatment (Figure 7E), corresponding to an increasing percentage of C_6_ volatiles (Figure 5C).

**Figure 8:**
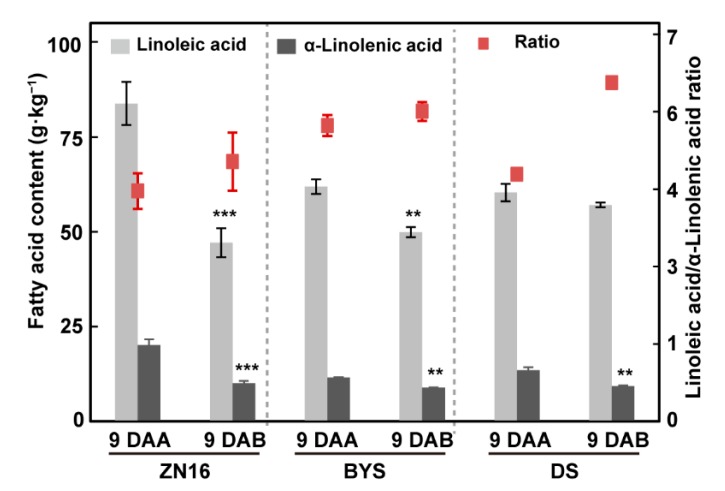
Analysis of linoleic acid and α-linolenic acid content and ratio after darkening treatment in cucumber fruits. Fatty acid (linoleic acid and α-linolenic acid) content was based on per unit dry weight in different cucumber fruits at 9 DAA/9 DAB. The red points represent the linoleic acid/α-linolenic acid ratio indicated by the Y-axis on the right. Bars represent means ± standard deviations (*n* = 3); data were analyzed by one-way ANOVA followed by Tukey’s test (*** *P* < 0.001, ** *P* < 0.01).

Linoleic acid and α-linolenic acid are precursors of (*E,Z*)-2,6-nonadienal and (*E*)-2-nonenal, respectively. Therefore, the ratio of linoleic acid to α-linolenic acid is an important indicator of cucumber fruit flavor [7]. After bagging treatment, both linoleic acid and α-linolenic acid contents were significantly decreased relative to those in non-bagged fruits, particularly for α-linolenic acid to a large extend, inducing higher linoleic/α-linolenic acid ratios in bagged fruits of “ZN16”, “BYS”, and “DS” (Figure 8), and suggesting a possible increase of “cucumber-like” flavor in bagged fruits.

## 4. Discussion

Cucumber is an economically important vegetable crop worldwide. It is native to the southern Asian continent [34] and can be divided into four geographic groups: the Indian group (mainly from India), Eurasian group (primarily from central and western Asia, Europe, and the United States), East Asian group (mainly from China, Korea, and Japan), and Xishuangbanna group cultivated in the Xishuangbanna region of tropical southwestern China [35]. Of the three genotypes used in this study, “ZN16” and “BYS” are identified morphologically as cultivated forms of the East Asian group, while “DS” comes from the Eurasian group. In general, cucumber fruit quality, including external, nutritional, and flavor characteristics, change significantly after bagging treatment.

### 4.1. Bagging Impacts Fruit Appearance Quality and Nutritional Characteristics

Chlorophyll and carotenoids are two major pigments in higher plants. Biosynthesis and function of the tetrapyrrole chlorophyll in plants occurs exclusively in plastids [36]. Carotenoids are lipophilic isoprenoid pigments that are synthesized by all photosynthetic organisms, including plants, and their production is tightly regulated by light [4]. Carotenoid biosynthesis in ripening tomato fruit is regulated by phytochrome-interacting factors [37] that are identical to those regulating carotenogenesis in Arabidopsis leaves in response to light signals [38]. Moreover, degraded chloroplasts/etioplasts without distinct grana stacking are observed in the exocarp of bagged fruit at 9 DAB [19]. Bagging treatment excluded light, resulting in degradation of plastids, thereby inducing a substantial reduction in pigment content and color change.

The concentration of ascorbate was reduced after darkening treatment. In general, ascorbate concentrations are regulated by light. For example, leaf ascorbate concentrations in *Arabidopsis thaliana* increase under high light intensities [39]. This response to light is associated with changes in the expression levels of several L-galactose pathway genes [20,39]. However, the effect of light on ascorbate content is considered to be greater in leaves than in ripening fruits. Seven days of shading to tomato (*Solanum lycopersicum*) decreases total ascorbate content by 50% in leaves and by merely 10% in fruits [20]. Among the last six steps of ascorbate biosynthesis, only two genes were down-regulated by long-term shading in red ripe fruits, compared to seven genes in leaves [20]. Moreover, concentration and accumulation rates of ascorbate in kiwifruit vary markedly between fruit genotypes and this is related to changes in gene expression [40]. In our study, ascorbate content was reduced by varying degrees in bagged fruit of different genotypes (from 4.5% in “ZN16” to 20.7% in “DS”).

Soluble solids content is one criterion used to measure the marketing grade of cucumber fruits. Thus, carbohydrate metabolism has a pivotal role in cucumber fruit. The primary sugars accumulated in cucumber fruits are glucose and fructose [41]. Under normal conditions, photosynthesis of cucumber (“ZN16”) fruit contributes merely 9.4% of the total carbohydrate required for its own growth [19], indicating that most of the assimilates in fruit come from the leaves. Consequently, the levels of glucose, fructose, and starch almost had no changes in bagged fruits compared with control fruits. Cellulose content is associated with fruit flesh crispness [42] and was decreased by 9.0%, 24.0%, and 16.1% in bagged “ZN16”, “BYS”, and “DS” fruits, respectively. The synthesis of cellulose microfibrils is catalyzed by cellulose synthase complexes containing six cellulose synthase genes, which are regulated by light conditions in Arabidopsis [43].

### 4.2. Bagging Would be Conducive to Accumulation of C_6_ Volatiles in Cucumber Fruit

Volatile C_6_ and C_9_ compounds (including aldehydes and alcohols) are important factors in fruit and vegetable flavor. Here, we report that bagging treatment of cucumber fruit led to remarkably higher accumulation of C_6_ aldehydes and C_6_ alcohols, such as *n*-hexanal and (*E*)-2-hexenal (except in “DS”), as well as 1-hexanol and (*E*)-2-hexen-1-ol. Previous research on the effects of darkening on volatile production in other fruit has been contradictory. On the one hand, a reduction in C_6_ aldehydes occurs in peaches and strawberries upon shading, respectively, with two-layered paper bags (black inner and brown outer paper) and 47% shading [8,44]. On the other hand, the levels of (*E*)-2-hexenal in peach fruit, as well as the formation of C_6_ compounds in grapefruit, are both significantly higher after bagging/shading treatment [9,17]. The starting point for volatile synthesis begins with carbon dioxide and photosynthesis, leading to the production of primary metabolic products. The reduction in photosynthesis caused by shading of entire strawberry plants reduces the amount of primary metabolic products produced by the plant and, in turn, provides fewer raw materials for volatile synthesis [44]. In our study, bagging was applied only to the cucumber fruit, and not the whole plant. Furthermore, most photosynthetic products in cucumber fruits come from the unfolding leaves. Darkening the fruits could, in turn, stimulate increased photosynthesis in leaves [19], thereby leading to higher accumulation of C_6_ aldehydes and alcohols in bagged fruits.

C_6_ and C_9_ compounds are produced via the LOX and HPL pathways [45]. LOX and HPL activities and expression of the corresponding encoded genes are regulated by growth and development stage, hormones, and adversities such as light, wounding, and cold and salt stress [13,16,46,47]. Expression levels of *Oe1LOX2*, *Oe2LOX2*, *Oe2LOX1*, and *OeHPL* in olive fruit [47,48] and *ZmLOX10* in maize [46] are depressed during growth in darkness, indicating light-dependent and circadian transcriptional regulation of these genes. Bagging peach fruit have significantly higher LOX and HPL enzyme activities than control fruit, although unaltered *LOX* transcripts and significantly up-regulated *PpHPL1* transcripts are observed [9]. 

In our study, bagging altered the microenvironment for fruit growth, thereby influencing the transcriptional and enzymatic levels of LOX and HPL. However, *CsLOX* and *CsHPL* family genes displayed different expression patterns in bagged fruits, possibly due to differences among cucumber genotypes. Therefore, the accumulation of both C_6_ aldehydes and alcohols in bagged fruits of “ZN16” and “BYS” (from the East Asian group) was probably due to increased enzymatic activity of 13-HPL, which catalyzes the formation of C_6_ aldehydes. Decreased enzyme activity of LOX in “DS” shaded fruits (from the Eurasian group) may limit the accumulation of C_6_ and C_9_ aldehydes even when accompanied by increased 13-HPL. That is to say, reduced LOX activity perhaps made a greater difference to lowering aldehyde content than increased 13-HPL activity. Furthermore, higher C_6_ and C_9_ alcohol content in “DS” 9 DAB fruits may result from improved 13-HPL activity. Nevertheless, (*E,Z*)-2,6-nonadienal/(*E*)-2-nonenal ratio and linoleic acid/α-linolenic acid ratio increased in all three genotypes after darkening, indicating probably stronger cucumber-like flavor after shading treatment.

## 5. Conclusions

This paper discusses the changing characteristics of the fruit color, nutritional quality and volatile compounds of three cucumber genotypes from different geographic groups after bagging treatment. Levels of chlorophyll, carotenoid and ascorbate were decreased to some extent by bagging. C_6_ aldehyde and alcohol compounds, (*E,Z*)-2,6-nonadienal/(*E*)-2-nonenal ratio, and linoleic/α-linolenic acid ratio enhanced largely through bagging treatment, accompanied by the variation of gene family expression and enzyme activity involved in lipoxygenase (LOX) and hydroperoxide lyase (HPL) pathways, suggesting that bagging is conducive to altering the exocarp color and improving volatile composition. These findings will be of great significance to clarify the formation mechanism of volatile compounds in fruit-environment interactions, and to provide reference for cultivation conditions to improve cucumber fruit flavor to a considerable degree.

## Figures and Tables

**Table 1:** Odor thresholds and aroma values of different cucumber fruits.

Compound	Odor Threshold (μg·kg^−1^)	Aroma Value						Flavor Description
		ZN16–9 DAA	ZN16–9 DAB	BYS–9 DAA	BYS–9 DAB	DS–9 DAA	DS–9 DAB	
(*E,Z*)-2,6-Nonadienal	0.01	9.95 × 10^4^ ± 4.25 × 10^3^	1.23 × 10^5^ ± 1.58 × 10^4^ ↑	8.49 × 10^4^ ± 9.0 × 10^3^	1.65 × 10^5^ ± 1.28 × 10^4^ ↑	2.27 × 10^5^ ± 9.7 × 10^3^	7.83 × 10^4^ ± 6.7 × 10^3^ ↓	Cucumber like
(*E*)-2-Nonenal	0.50	1.37 × 10^3^ ± 3.08×10^2^	1.05 × 10^3^ ±1.54 × 10^2^	1.48 × 10^3^ ± 1.40 × 10^2^	2.05 × 10^3^ ± 5.18 × 10^2^ ↑	4.33 × 10^3^ ± 4.94 × 10^2^	6.90 × 10^2^ ± 72 ↓	Fatty, green
Nonanal	1.00	69.45 ± 4.91	65.69 ±15.50	97.58 ± 23.19	101.82 ± 18.36	41.96 ± 20.35	43.33 ± 3.97	Orange like
*n*-Hexanal	4.50	17.78 ± 2.22	83.56 ± 13.11 ↑	28.67 ± 0.22	102.00 ± 5.11 ↑	99.33 ± 18.44	41.33 ± 9.78 ↓	Green grass
(*E*)-2-Hexenal	17.00	7.00 ± 1.47	18.47 ± 2.06 ↑	4.88 ±0.24	16.29 ± 1.53 ↑	17.06 ± 2.94	12.65 ± 2.24 ↓	Apple like
Propanal	9.50–37.00	0.55–2.12	5.08–19.78 ↑	0.58–2.28	4.18–16.28 ↑	7.00–27.26	3.67–14.31 ↓	Stimulate
(*E,E*)-2,4-Heptadienal	10.00	10.28 ± 1.67	25.13 ± 6.56 ↑	8.00 ± 1.10	27.82 ± 5.89 ↑	10.20 ± 1.54	11.05 ± 2.19	Fresh
(*Z*)-2-Heptenal	0.80	26.25 ± 7.50	76.25 ± 23.75 ↑	34.93 ± 1.25	101.25 ± 8.75 ↑	ND	48.75 ± 16.25 ↑	Fresh
(*E,Z*)-3,6-Nonadien-1-ol	10.00	ND	11.18 ± 1.59 ↑	ND	ND	ND	14.02 ± 0.46 ↑	Musky
1-Hexanol	250.00	0.27 ± 0.07	1.24 ± 0.28 ↑	0.12 ± 0.03	1.30 ± 0.43 ↑	0.51 ± 0.34	2.71 ± 0.31 ↑	Strawberry, fresh

Data represent the mean ± standard deviations of three independent biological determinations. Aroma value is calculated by dividing the content of volatile substances (see Supplementary Table S1) by their odor threshold content. Up and down arrows indicate relative increase or decrease compared with control fruit, respectively. DAA, days after anthesis, DAB, days after bagging.

## Supplementary Materials

The following are available online at https://www.mdpi.com/2304-8158/9/3/294/s1. Table S1: Identification and quantification of volatiles in different cucumber fruits, Table S2: Accession numbers of sequences used for reverse-transcription quantitative PCR analysis.
