# Supplementary material for: Comprehensive Characterization of Fruit Volatiles and Nutritional Quality of Three Cucumber (Cucumis sativus L.) Genotypes from Different Geographic Groups after Bagging Treatment
Source: Foods. 2020 Mar 5;9(3):294. doi: 10.3390/foods9030294 (PMC7143270; doi:10.3390/foods9030294)
Supplement: Supplementary file 1 [file foods-09-00294-s001.pdf]

Table S1. Identification and quantification of volatiles in different cucumber fruits (mg·kg^{-1}).

	Compound name	ZN16		BYS		DS	
		**9 DAA**	**9 DAB**	**9 DAA**	**9 DAB**	**9 DAA**	**9 DAB**
Aldehydes	Hexanal	0.080 ± 0.010	0.376 ± 0.059	0.129 ± 0.001	0.459 ± 0.023	0.447 ± 0.083	0.186 ± 0.044
	(*E*)-2-Hexenal	0.119 ± 0.025	0.314 ± 0.035	0.083 ± 0.004	0.277 ± 0.026	0.290 ± 0.050	0.215 ± 0.038
	Sub total C6 aldehyde	***0.223±0.040***	***0.690 ±0.089***	***0.211 ± 0.003***	***0.736 ± 0.013***	***0.737 ± 0.133***	***0.400 ± 0.080***
	Nonanal	0.069 ± 0.005	0.066 ± 0.015	0.098 ± 0.023	0.102 ± 0.018	0.042 ± 0.020	0.043 ± 0.004
	(*E*)-2-Nonenal	0.683 ± 0.154	0.524 ± 0.077	0.740 ± 0.070	1.026 ± 0.259	2.167 ± 0.247	0.345 ± 0.036
	(*Z*)-6-Nonenal	0.189 ± 0.030	0.172 ± 0.035	0.136 ± 0.002	0.204 ± 0.036	ND	0.056 ± 0.005
	(*E*,*Z*)-2,6-Nonadienal	0.995 ± 0.043	1.229 ± 0.158	0.849 ± 0.090	1.648 ± 0.128	2.273 ± 0.097	0.783 ± 0.067
	Sub total C9 aldehyde	***1.944± 0.296***	***1.991 ± 0.247***	***1.823 ± 0.046***	***2.853 ± 0.020***	***4.665 ± 0.435***	***1.228 ± 0.112***
	(*E*,*E*)-2,4-Heptadienal	0.103 ± 0.017	0.251 ± 0.066	0.080 ± 0.011	0.278 ± 0.059	0.102 ± 0.015	0.110 ± 0.022
	(*E*)-2-Heptenal	ND	ND	ND	ND	0.070 ± 0.015	ND
	(*Z*)-2-Heptenal	0.021 ± 0.006	0.061 ± 0.019	0.028 ± 0.001	0.081 ± 0.007	ND	0.039 ± 0.013
	Propanal	0.020 ± 0.006	0.188 ± 0.004	0.022 ± 0.001	0.155 ± 0.008	0.259 ± 0.017	0.136 ± 0.057
	(*E*)-2-Octenal	0.015 ± 0.004	0.027 ± 0.007	0.016 ± 0.001	0.034 ± 0.003	0.041 ± 0.014	
	(*Z*)-9,17-Octadecadienal	0.236 ± 0.055	0.109 ± 0.031	0.085 ± 0.041	0.046 ± 0.029	0.035 ± 0.024	0.012 ± 0.002
	(*Z*)-9-Octadecenal	0.011 ± 0.001	ND	ND	ND	ND	ND
	cis,*cis*,*cis*-7,10,13-Hexadecatrienal	ND	ND	0.045 ± 0.025	ND	ND	ND
	Heptanal	ND	0.013 ± 0.003	0.011 ± 0.000	0.016 ± 0.004	0.009 ± 0.001	0.010 ± 0.002
	Hexadecanal	0.529 ± 0.060	0.367 ± 0.111	0.306 ± 0.121	0.214 ± 0.033	ND	ND
	Tetradecanal	0.073 ± 0.015	ND			0.148 ± 0.102	0.112 ± 0.001
(Continued)							
	Undecanal	0.020 ± 0.002	0.011 ± 0.002	0.014 ± 0.008	0.007 ± 0.002	ND	ND

	Benzaldehyde	ND	ND	0.010 ± 0.003		ND	0.066 ± 0.022
Alcohols	1-Hexanol	0.068 ± 0.018	0.310 ± 0.070	0.030 ± 0.007	0.325 ± 0.108	0.128 ± 0.085	0.677 ± 0.078
	(*E*)-2-Hexen-1-ol	ND	ND	ND	ND	ND	0.045 ± 0.002
	Sub total C6 alcohol	***0.068 ± 0.018***	***0.310 ± 0.070***	***0.030 ± 0.007***	***0.325 ± 0.108***	***0.128 ± 0.085***	***0.765 ± 0.036***
	(*E*,*Z*)-2,6-Nonadien-1-ol	0.174 ± 0.078	0.100 ± 0.029	0.125 ± 0.005	0.404 ± 0.094	0.160 ± 0.038	1.039 ± 0.255
	(*E*,*Z*)-3,6-Nonadien-1-ol	ND	0.112 ± 0.016	ND	ND	ND	0.140 ± 0.005
	(*E*)-2-Nonen-1-ol	0.083 ± 0.031	ND	0.126 ± 0.021	ND	ND	ND
	(*Z*)-6-Nonen-1-ol	0.266 ± 0.143	0.118 ± 0.036	0.156 ± 0.014	ND	ND	1.123 ± 0.155
	(*E*)-3-Nonen-1-ol	ND	ND	ND	ND	ND	0.028 ± 0.002
	Sub total C9 alcohol	***0.523 ± 0.252***	***0.290 ± 0.031***	***0.407 ± 0.030***	***0.404 ± 0.094***	***0.160 ± 0.038***	***2.469 ± 0.279***
	1-Heptanol	ND	ND	0.010 ± 0.001	0.017 ± 0.004	0.009 ± 0.005	0.028 ± 0.011
	1-Octanol	ND	ND	0.161 ± 0.102	ND	ND	ND
	1-Octen-3-ol	0.012 ± 0.003	0.036 ± 0.009	0.018 ± 0.001	0.040 ± 0.006	0.031 ± 0.010	0.041 ± 0.003
	Octanol	ND	ND	ND	ND	ND	0.109 ± 0.006
	(*E*)-2-Octen-1-ol	ND	0.041 ± 0.007	ND	ND	ND	0.756 ± 0.167
	3-Ethyl-3-undecanol	ND	ND	ND	ND	0.139 ± 0.055	ND
	(*Z*,*Z*,*Z*)-9,12,15-Octadecatrien-1-ol	0.297 ± 0.079	0.078 ± 0.009	ND	ND	ND	ND
	2,5-Dimethylcyclohexanol	ND	ND	ND	ND	0.026 ± 0.013	ND
Ketones	2,5-Octanedione	0.025 ± 0.004	0.035 ± 0.008	0.024 ± 0.001	0.042 ± 0.005	0.023 ± 0.004	0.025 ± 0.002
	2,5,9-Trimethylcycloundeca-4,8-dienone	ND	ND	ND	ND	0.038 ± 0.002	ND
	3-Buten-2-one, 4-(2,6,6-trimethyl-1-cyclohexen-1-yl)	0.020 ± 0.001	0.009 ± 0.003	0.006 ± 0.000	ND	ND	ND
(Continued)							
	3,5-Octadien-2-one	0.034 ± 0.004	0.221 ± 0.048	ND	0.212 ± 0.011	0.065 ± 0.017	0.061 ± 0.008
	3-Isopropyl-5-methylhexan-2-one	0.017 ± 0.006	0.079 ± 0.015	0.021 ± 0.006	ND	0.141 ± 0.037	ND
	7-Oxabicyclo[2.2.1]hept-5-en-2-one	ND	0.050 ± 0.012	ND	0.057 ± 0.006	ND	ND

Hydrocarbons	(*Z*)-5-Tridecene	ND	ND	ND	ND	0.116 ± 0.042	ND
	(*E*)-7-Tetradecene	ND	ND	ND	ND	ND	0.167 ± 0.020
	d-Limonene	ND	ND	ND	0.050 ± 0.011	ND	ND
	α-Caryophyllene	0.039 ± 0.009	0.016 ± 0.003	0.046 ± 0.008	0.018 ± 0.004	0.017 ± 0.001	0.009 ± 0.001
	Caryophyllene oxide	0.042 ± 0.015	0.027 ± 0.005	0.042 ± 0.005	0.030 ± 0.004	0.031 ± 0.011	ND
	β-Caryophyllene	0.042 ± 0.008	0.012 ± 0.002	0.071 ± 0.012	0.013 ± 0.004	ND	ND
	3-Ethyl-2-methyl-1-heptene	ND	ND	0.025 ± 0.008	ND	ND	ND
	5,10-Dioxatricyclo[7.1.0.0(4,6)] decane	ND	ND	ND	ND	0.016 ± 0.005	ND
	2-Dodecyne	ND	ND	0.035 ± 0.007	ND	ND	ND
	1,1-dimethyl-2-(2-methyl-2-propenyl)-Cyclopropane	ND	ND	ND	ND	0.103 ± 0.007	ND
	2-Nonyne	0.045 ± 0.003	ND	ND	ND	ND	ND
Others	Methyl salicylate	ND	ND	0.051 ± 0.005	ND	ND	ND
	Tetrahydro-1H,3H-furo[3,4-c]furan	ND	ND	ND	ND	0.048 ± 0.013	ND
	2-Pentylfuran	0.009 ± 0.000	0.020 ± 0.005	0.010 ± 0.001	0.020 ± 0.004	0.017 ± 0.006	0.018 ± 0.002

Data represent the mean ± standard deviations of three independent biological determinations. DAA, days after anthesis; DAB, days after bagging; ND, not detected.

Table S2. Accession numbers of sequences used for reverse-transcription quantitative PCR analysis.

Lipoxygenase gene family		Hydroperoxide lyase gene family	
**Gene name**	**Accession number**	**Gene name**	**Accession number**
CsLOX1	Csa2G023880	*CsHPL1*	Csa7G041960
CsLOX2	Csa2G023920	*CsHPL2*	Csa7G075590
CsLOX4	Csa2G028500	*CsHPL3*	Csa7G075600
CsLOX8	Csa2G023840		
CsLOX9	Csa6G424000		
CsLOX10	Csa2G023340		
CsLOX16	Csa4G286960		
CsLOX17	Csa7G374620		
CsLOX19	Csa4G288610		
CsLOX20	Csa4G288080		
CsLOX22	Csa7G449420		
CsLOX23	Csa5G590080		
